# Translation and Validation of the Ureteral Stent Symptoms Questionnaire in Urdu

**DOI:** 10.7759/cureus.27764

**Published:** 2022-08-08

**Authors:** Kaleem K Mirani, M. H Ather

**Affiliations:** 1 Urology, Aga Khan University, Karachi, PAK

**Keywords:** validation, reliability, urdu, ussq, ureteral stents

## Abstract

Introduction

In endourology, ureteric stenting is a common procedure, and stent placement is not without adverse health consequences. A ureteric stent symptoms questionnaire (USSQ) was devised to objectively evaluate the symptoms related to it. The original questionnaire is in English and translated into various languages worldwide. We translated the questionnaire into Urdu and validated it in an Urdu-speaking population with a stent in situ.

Materials and methods

The English version of USSQ was translated and back-translated by experts in both languages. Content validity was checked by sending Urdu version to five experts, and their scores were used to calculate the content validity index. The final version was filled by patients with stents on three different occasions, two with stents in situ at one and two weeks post stent placement, and a third time two weeks after stent removal. Discriminant validity was checked by filling of USSQ by 64 healthy individuals. Statistical analysis was done with mean and standard deviation, Cronbach’s alpha, Spearman’s coefficient, and paired sample t-test.

Results

A total of 64 patients filled the complete questionnaire at all times with mean age of 35.31 ± 8.853. All subdomains of USSQ have significant drop in scores with stent in situ in comparison to post stent removal. Reliability was checked by Cronbach’s alpha in all subdomains (71.5-91.1) and test-retest reliability by Spearman’s coefficient (80.5-94.7). Symptoms change in stent in situ with post stent removal checked with paired sample t-test with a p-value of <0.005 in all domains, except body pain. Discriminant validity was checked with healthy controls, and a p-value of <0.005 was found in all subdomains of USSQ, except pain.

Conclusion

The Urdu version of the USSQ is a reliable and valid instrument that can be used in clinical practice and future research in an Urdu-speaking population.

## Introduction

Over the past century, minimally invasive surgery has advanced significantly, and it is now making its way into urology [1]. Routine endourological interventions, such as ureteral stenting, are one of the numerous urological treatments [2]. Common reasons for ureteral stent placement in urological surgery include preserving kidney function, relieving ureteral blockage, treating remaining stone pieces, healing mucosal injury or edema, treating hematomas, preventing urine extravasation, and relieving discomfort [3, 4]. Even though ureteral stents are used to treat serious conditions, there are still issues with them. Lower urinary tract symptoms, hematuria, flank pain, body aches, sexual dysfunction, and poor work performance are all common side effects of ureteral stent placement that also affect the quality of life. The main causes of ureteral stent-related symptoms are trigonal and renal irritation, vesico-renal reflux, the position of stent ends, stent length, stent diameter, and stent material. To address these issues, numerous modifications have been made. [5].

To assess and examine the ureteral stent-related symptoms, Joshi et al. developed and validated a questionnaire in 2003 and named it the ureteral stent symptom questionnaire (USSQ) [6]. It's a self-administered, multi-tiered tool with six sections and one question on the overall quality of life. The six sections are about urinary symptoms: bodily pain, general health, work performance, sexual issues, and additional problems [7]. Each section contains several questions, of which the answers are summed to provide an index score. Urinary symptoms domain (11-56 scores), body pain (2-43 scores), general health (4-28 scores), work performance (5-25 scores), sexual matters (1-12 scores), and additional problems (5-17 scores). Each of these categories alone has a considerable impact, and when the total effects are added up, they have an impact on quality of life [8]. This questionnaire has been translated and validated in many international and regional languages. Baghel et al. translated USSQ into Hindi and found good validity and reliability (Cronbach's α coefficient >0.44 and Spearman's correlation coefficient >0.44) and found high discriminant validity between patients with stent and healthy controls (p <0.005) [9]. Similarly, the intraclass correlation coefficient (ICC) of the USSQ domains ranged from 0.4 to 0.87 in a Persian validation study [10].

Urdu is the first language of many people in southeast Asia and is understood by many others in this part of the world. International prostatic symptoms score (IPSS) was translated and validated in Urdu, and now there's a need to translate the USSQ into the Urdu language so that it can be implemented in those parts of the world where Urdu is spoken as one of the major languages.

The original USSQ is in the English language. Its usage in the non-English population has prompted translations into many different languages worldwide. However, there is no translation available in Urdu, a language that is widely spoken and understood by people in Pakistan and many parts of Southeast Asia. Moreover, it needs to be validated before the usage of a translated version in clinical practice. Thus, we planned and conducted a study to translate and validate USSQ for patients who understand Urdu. Scoring will help to better understand the stent-related symptoms in the patient's language as its a self-administered questionnaire.

## Materials and methods

Our study was conducted from June 2021 to May 2022 at tertiary care hospital, Aga Khan University Hospital, in Karachi, Pakistan. The study was approved by the Institutional Ethics Committee with Ethical review committee number 2021-6241-18276. Two experts with proficiency in English and Urdu (one urologist with a fellowship degree in urology and one public health expert) individually translated the original English version of the ureteric stent symptoms questionnaire into Urdu. Backward translation from Urdu to English by two other experts to ensure that during the reverse translation, there was no major discrepancy between the two versions. To further refine the translation, a pilot study among 10 patients (five males and five females) was done by carrying out cognitive debriefing interviews to assess understandability and to highlight any discrepancies with the original version. The final Urdu version of the ureteric stent symptoms questionnaire was developed and sent to five health experts for content validity, and after receiving results from them it was used for the study population. The sample size was calculated by PASS11 software (NCSS Statistical Software, Kaysville, Utah). We required a minimum of 61 patients with a JJ stent in situ with an inflation of 10%, with an anticipated intraclass correlation of 0.6, a level of significance of 5%, and a power of 80%. 

Adult patients from age 18-80 years, who can read and understand Urdu, who underwent ureteral stent (DJ stent) placement after percutaneous nephrolithotomy, semi-rigid or flexible ureteroscopy in the study period at our hospital were included in the study after obtaining written informed consent. The exclusion criteria included 1) patients with bilateral stents in situ, 2) patients with recurrent urinary tract infections, lower urinary tract symptoms before stent insertion, chronic prostatitis, pelvic pain syndrome, 3) Pregnant females, 4) patients with residual stone, 5) patients with neurological/psychiatric condition precluding the use of the questionnaire, 6) some severe other diseases (such as unstable angina, congestive heart failure, signiﬁcant renal or hepatic dysfunction) affecting the quality of life, and 7) patients who refused to participate.

A total of 64 patients, male (n=44) and female (n=20), were enrolled in the study fulfilling the inclusion and exclusion criteria. The 6Fr Percuflex DJ stent with hydroplus coating (Boston Scientific, Marlborough, Massachusetts) was placed after the procedure. Position and coiling of the DJ stent were confirmed by fluoroscopy at the end of the procedure in all cases, and images were attached to the file of the patient. All patients were advised analgesics for one week on a regular basis and then as per need basis. All patients were given tamsulosin 0.4mg as standard practice to decrease the stent-related symptoms till the stent was in situ. All patients visited outpatient urology clinics one week after the procedure with a fresh kidney, ureter, and bladder (KUB) X-ray (with bowel preparation) to look for residual stones. All patients who had already consented to the study and signed the consent form then filled out the English and Urdu version of USSQ. Data for demographics, including age, sex, comorbid conditions, marital status, and education level collected by the primary investigator on predefined proforma. The patients visited the outpatient clinic again two weeks post-stent placement and filled the USSQ to analyze the internal consistency. All patients again filled the Urdu version of USSQ two weeks after DJ stent removal. Sixty-four healthy individuals who presented to our hospital in outpatient clinics of dermatology filled the USSQ, and this data was used in validation.

All analyses were conducted using SPSS 21.0 (IBM Inc., Armonk, New York). Descriptive for quantitative variables will be reported as mean ± SD, and qualitative variables will be reported as frequency and percentages. Psychometric analyses were used to check the internal consistency, test-retest reliability, and validity. Internal consistency was evaluated with Cronbach’s alpha, and ≥0.70 was considered to be a good level of internal consistency. Test-retest reliability was evaluated by comparing scores one week after stent placement and two weeks after stent placement using Pearson correlation coefﬁcient, and the minimal acceptable level will be deﬁned as 0.70. Content validity was established by measuring the content validity index from scores given by five health experts for every item. Discriminant validity was established by paired t-test by comparing scores of the Urdu version of USSQ filled at one week post stent placement with age and sex-matched 64 controls who did not have any urological symptoms and filled the USSQ Urdu version. A p-value of <0.05 was considered significant.

## Results

Based on responses from five experts, content validity index (CVI) was calculated at 0.87. After that, USSQ was applied to cases and controls who agreed to participate in the study and signed the informed consent form. After removing the cases and controls with incomplete forms and cases that failed to follow till the required time, 64 cases and 64 controls were included in the final analysis. The basic demographics of the cases are shown in Table 1. The mean age was 35.31 ± 8.85. The number of male participants was 44 (68.8%), while 20 participants were female (31.2%). Five (7.8%) patients had an education level below matriculation, five (7.8%) patients were matriculation or O level passed, and 54 (84.4%) patients had a qualification above matriculation. Out of 64 participants, 56 (87.5%) were married, and eight (12.5%) were single/not in a relationship. The mean urinary symptom score of 30.53 ± 2.03 at one week post stent placement and 11.67 ± 0.79 at two weeks post stent removal. Body pain score also reduced from 18.26 ± 1.54 with the stent in situ to 12.66 ± 2.88 two weeks post stent removal, and all other subparameters also showed a decrease in scores after stent removal, as shown in Table 2.

**Table 1:** Demographics

Demographics	Total % /S.D. (n=64)
Age	35.31 ± 8.853
Gender	
Male	44 (68.8%)
Female	20 (31.2%)
Education	
Below matriculation/O level	5 (7.8%)
Matriculation/O level	5 (7.8%)
Above matriculation/O level	54 (84.4%)
Marital status	
Single	8 (12.5%)
Married	56 (87.5%)
Work status	
Full-time job	39 (60.94%)
Part-time job	08 (12.5%)
Unemployed	16 (25%)
Student	01 (1.56%)

**Table 2:** USSQ scores one week post DJ placement, two weeks post DJ placement, and two weeks post DJ removal USSQ - ureteric stent symptoms questionnaire, QoL - quality of life

Domain	1 week post DJ stent placement	2 weeks post DJ stent placement	2 weeks post DJ stent removal
Urinary symptoms	30.53 (2.03)	30.15 (2.03)	11.67 (0.79)
Body pain	18.26 (1.54)	18.09 (1.54)	12.66 (2.88)
General health	11.40 (1.92)	11.14 (1.85)	6.14 (0.53)
Work performance	9.78 (1.14)	9.68 (1.10)	5.50 (0.83)
Sexual matters	4.86 (0.89)	4.74 (0.84)	2.11 (0.43)
Global QoL	3.70 (0.65)	3.46 (0.66)	1.86 (0.49)

Reliability of the ureteral stent symptoms questionnaire was checked by Cronbach’s α values of each domain at one week post stent placement and two weeks post stent placement. Test-retest reliability was checked by Spearman’s coefficient values of these two Cronbach’s alpha at different levels, i.e., at one week post stent placement and two weeks post stent placement, as shown in Table 3. Cronbach’s α level of more than 0.70 is considered significant, and Spearman’s coefficient of more than 0.80 is considered significant. All values of Cronbach’s alpha decreased from one to two weeks post stent placement which means all parameters were worse at one week post stent placement and slightly improved at two weeks post stent placement. A positive correlation was found between the two domains by Spearman’s coefficient, as shown in Table 3. Reliability is further strengthened by calculating Spearman’s coefficient at two different levels of stent placement, i.e., at one week and two weeks post stent placement, as shown in Table 4.

**Table 3:** Reliability of the ureteral stent symptoms questionnaire Urdu translation QoL - quality of life

Domain	Cronbach’s alpha (1 week post stent placement)	Cronbach’s alpha (2 weeks post stent placement)	Test-retest reliability (Spearman’s correlation coefficient)
Urinary symptoms	91.1	89.0	94.7
Body pain	83.9	75.0	87.3
General health	77.0	74.5	90.9
Work performance	71.5	69.1	91.4
Sexual matters	79.9	71.9	91.4
Global QoL	Not applicable	Not applicable	80.5

**Table 4:** Corelation of USSQ domains with stent in situ at two different levels i.e., at one week post stent placement and two weeks post stent placement USSQ - ureteric stent symptoms questionnaire, QoL - quality of life

Domain			USSQ at 1 week						
			Urinary symptoms		Body pain	General health	Work performance	Sexual health	Global QoL
USSQ at 2 weeks	Urinary symptoms		1/1						
	Body pain		0.54/0.23		1/1				
	General health		0.89/0.96		0.60/0.39	1/1			
	Work performance		0.72/0.66		0.74/0.81	0.23/0.68	1/1		
	Sexual health		0.50/0.53		0.45/0.85	0.39/0.54	0.95/0.84	1/1	
	Global QoL		0.97/0.79		0.84/0.36	0.94/0.83	0.35/0.25	0.37/0.56	1/1

The sensitivity to change in symptoms was analyzed at two different levels with the stent in situ and post stent removal. Paired sample t-test was run between each domain score at one week post stent placement with two weeks post stent removal, and a p-value of <0.005 was considered significant. A paired sample t-test was also run between each domain score at two weeks post stent placement and post stent removal, and a p-value of <0.005 was considered significant. In our study, we found significant p-value in all domains at both times, i.e., one week with the stent in situ and post stent removal and two weeks with the stent in situ and post stent removal, except for bodily pain where the p-value was more than 0.005 as shown in Table 5.

**Table 5:** Sensitivity to change of the Urdu translation of USSQ (paired sample t-test) USSQ - ureteric stent symptoms questionnaire, QoL - quality of life

Domain	1 week post stent placement versus post DJ stent removal	2 weeks post DJ stent placement versus post DJ stent removal
Urinary symptoms	<0.001	<0.001
Body pain	0.057	0.057
General health	<0.001	<0.001
Work performance	<0.001	<0.001
Sexual matters	<0.001	<0.001
Global QoL	<0.001	<0.001

Discriminant validity of the questionnaire was assessed by age, sex, and body mass index-matched controls with patients with a stent in situ, and it is a valid measure between patients and healthy controls who don’t have a history of stent placement or lower urinary tract symptoms. Paired sample t-test run between the case with a stent in situ and healthy controls and a p-value of <0.005 was considered significant. In our study, controls have significantly lower symptoms in all domains than patients with a stent in situ, and the p-value is lower than 0.005 in all domains except in body pain, where the p-value was 0.15, as shown in Table 6. This p-value insignificance in body pain may be due to controls that were also present in some other clinics (dermatology clinic) at the same hospital with no urological symptoms.

**Table 6:** Discriminant validity of Urdu translation of USSQ (paired-samples t-test) USSQ - ureteric stent symptoms questionnaire, QoL - quality of life

Domain	1 week post stent placement	Controls	p-value
Urinary symptoms	30.53 (2.03)	11.50 (0.81)	<0.001
Body pain	18.26 (1.54)	13.5 (2.12)	0.15
General health	11.40 (1.92)	6.10 (0.44)	<0.001
Work performance	9.78 (1.14)	5.10 (0.40)	<0.001
Sexual matters	4.86 (0.89)	2.07 (0.27)	<0.001
Global QoL	3.70 (0.65)	1.82 (0.45)	<0.001

## Discussion

The first ureteral stent was implanted more than 50 years ago, and as endourology has advanced, so has the usage of ureteral stents [2, 11]. The increased use of a stent following endourological procedure is necessary, but on the other hand, these stents are not inert instruments and have their own morbidity and, in turn, affect the quality of life [12]. This bothersome and poor quality of life needs improvement in stent material, stent coiling and positioning of the stent, and diameter of the stent [13-15]. To unbiasedly evaluate the symptoms brought on by stents, Joshi et al. developed and validated the USSQ in 2003. It is a multi-tiered tool that assesses the morbidity associated with stents across six categories, three of which are peculiar to stents (urinary symptoms, bodily pain, and additional problems), while three others are connected to more general issues (general health, work performance, and sexual matters). These six domains are divided into 42 questions, and one question on the global quality of life [7].

Hindi, French, German, Spanish, and Arabic are just some of the languages in which the original USSQ has already been validated [9, 16-19]. In our study, the mean age of patients was 35.31 ± 8.85: older than in a Persian study [10] population with a mean age of 33 years, and comparable to a Hindi validation study [9] in which the median age was 35 years. All our participants (cases as well as controls) speak Urdu as their first language, and they can read and understand Urdu. Most of our patients were also able to read and understand English, and in very few cases and controls, the help of relatives was taken to fill the English version of the questionnaire. In our study, not a single case or control need help from the primary author in filling the questionnaire. Our findings demonstrate that the USSQ in Urdu is a viable and accurate tool for measuring a variety of stent-related symptoms in the Urdu-speaking community. These results show that the cross-cultural validity of this questionnaire is satisfactory and it may well also be applicable to other languages and cultures. Content validity of the Urdu version of the USSQ was promising according to the modified kappa value of 0.87. Mean and standard deviation calculated for all sub-domains at one week and two weeks post stent placement and then two weeks post stent removal and found that there was no significant change in any domains with the stent in situ at two different intervals, but found a significant difference in each domain post stent removal as shown in Table 2. Internal consistencies were found to be satisfactory for most domains (Table 3), suggesting its reliability. The good internal consistency recorded for the Urdu version (71.5-91.1) was consistent with previous studies on English, Spanish, Italian and Korean versions, which reported Cronbach’s alphas to be in the range of 0.60-0.96, 0.73-0.85, 0.73-0.83, and 0.37-0.92, respectively [7, 18, 20-21]. While the original USSQ study [7] and Italian study [20] put a four week interval to avoid recall bias in assessing test-retest reliability, we assessed it with a two week interval as we believe that recall bias from a relatively shorter interval is minimal. Test-retest reliability in our study was good (Spearman’s Coefficient 80.5-94.7) and higher than the Spanish [18], Italian [20], and Korean [21] versions, with Spearman correlation coefficients above 0.6 for Spanish [18] and Korean [21] versions and 0.35-0.72 for the Italian [20] version. Moderate to strong correlation was found between sub-domains of USSQ at one and two weeks with the stent in situ, and a very strong correlation was found between urinary symptoms and general health, as shown in Table 4. As shown in Table 4, there is a weak correlation between work performance and global quality of life; the reason behind this was that female patients in our part of the world usually don’t have a full-time job, as also in our study, only three out of 20 female patients had a full-time job. Also, in our study, a weak correlation was found between sexual activity and general health in the first week post stent placement, and it may be due to patients avoiding sexual activity in the first week post-surgery. This is also supported by a moderate correlation between sexual activity and general health two weeks post-surgery.

We found in our study significant change in all sub-domains with a p-value of less than 0.005 in patients with the stent in situ at one and two weeks with two weeks post stent removal, except in the body pain sub-domain, as shown in Table 5. The reason behind the insignificant difference in pain may be due to our routine practice is to prescribe medications during the first week post stent placement and use of analgesics as per need basis afterward. We also compare all subdomains of USSQ between patients with the stent in situ at one week post stent placement with healthy controls who presented in a dermatology clinic with no urological issues at that moment. Discriminant validity was calculated between these two groups and found a significant difference with a p-value of <0.005 in all sub-domains except in pain, as shown in Table 6. This insignificant difference in pain may be due to the control being patients as well, although non-urological, and may have some pain due to other causes. This great capacity to identify cases is indicated by the substantial p-value between patients and controls. Additionally, the linguistic validation studies for Hindi, Korean, and Spanish showed comparable findings. Giannarini et al., in contrast, evaluated the convergent validity by examining connections between the IPSS and urine symptoms and the visual analog scale and the bodily pain domain [20].

One of the drawbacks of our study was the inclusion of patients in the control group who had no prior urological history and who were not entirely healthy due to their hospital visits for dermatological issues. A better control group would have been patients who underwent the same procedure as the case but without the placement of stents. In our study, convergent validity was not calculated. In addition, or study had a small sample size. The amount of water consumed, heat exposure, and the amount of exercise performed may have an impact on the symptoms; however, these factors were not controlled in this study.

## Conclusions

The translated USSQ is a valid and reliable tool for assessing the symptoms of stents in the Urdu-speaking community. This will make it easier to objectively describe symptoms when a stent is in place and may also make it easier to evaluate symptoms when a variety of stent types are used in the first language of the Urdu-speaking community. This version may also be used in comparison studies and future research on stent-related symptoms.
